# Human Alveolar Epithelial Cells Attenuate Pulmonary Microvascular Endothelial Cell Permeability under Septic Conditions

**DOI:** 10.1371/journal.pone.0055311

**Published:** 2013-02-05

**Authors:** Lefeng Wang, Ravi Taneja, Wei Wang, Li-Juan Yao, Ruud A. W. Veldhuizen, Sean E. Gill, Dalilah Fortin, Richard Inculet, Richard Malthaner, Sanjay Mehta

**Affiliations:** 1 Centre for Critical Illness Research, Division of Respirology, Lawson Health Research Institute, London Health Sciences Center, London, Ontario, Canada; 2 Department of Medicine, Schulich Faculty of Medicine and Dentistry, Western University, London, Ontario, Canada; 3 Department of Anesthesia, Schulich Faculty of Medicine and Dentistry, Western University, London, Ontario, Canada; 4 Department of Critical Care Medicine, Schulich Faculty of Medicine and Dentistry, Western University, London, Ontario, Canada; 5 Department of Thoracic Surgery, Schulich Faculty of Medicine and Dentistry, Western University, London, Ontario, Canada; University of Nebraska Medical Center, United States of America

## Abstract

Acute lung injury (ALI) and its most severe form, acute respiratory distress syndrome (ARDS), are characterised by high-protein pulmonary edema and severe hypoxaemic respiratory failure due to increased permeability of pulmonary microvascular endothelial cells (PMVEC). Alveolar epithelial cells (AEC) contribute importantly to normal alveolar function, and AEC dysfunction in ALI/ARDS is associated with worse outcomes. We hypothesized that AEC can modulate human PMVEC barrier function, and investigated the effects of AEC presence on human PMVEC barrier under septic conditions in vitro. PMVEC isolated from human lung were treated in vitro with septic stimulation (lipopolysaccharide [LPS], a mixture of clinically-relevant cytokines [cytomix], or plasma from patients with severe sepsis), and the trans-PMVEC leak of Evans Blue dye-labeled albumin assessed. PMVEC septic responses were compared in the presence/absence of co-cultured A549 epithelial cell line or primary human AEC. Septic stimulation with LPS, cytomix, or septic plasma induced marked PMVEC hyper-permeability (10.2±1.8, 8.9±2.2, and 3.7±0.2 fold-increase vs. control, respectively, p<0.01 for all). The presence of A549 cells or primary human AEC in a non-contact co-culture model attenuated septic PMVEC hyper-permeability by 39±4% to 100±3%, depending on the septic stimulation (p<0.05). Septic PMVEC hyper-permeability was also attenuated following treatment with culture medium conditioned by previous incubation with either naïve or cytomix-treated A549 cells (p<0.05), and this protective effect of A549 cell-conditioned medium was both heat-stable and transferable following lipid extraction. Cytomix-stimulated PMN-dependent PMVEC hyper-permeability and trans-PMVEC PMN migration were also inhibited in the presence of A549 cells or A549 cell-conditioned medium (p<0.05). Human AEC appear to protect human PMVEC barrier function under septic conditions in vitro, through release of a soluble mediator(s), which are at least partly lipid in nature. This study suggests a scientific and potential clinical therapeutic importance of epithelial-endothelial cross talk in maintaining alveolar integrity in ALI/ARDS.

## Introduction

Acute lung injury (ALI) and its most severe form, acute respiratory distress syndrome (ARDS), remain major causes of morbidity and mortality in critically ill patients. ALI/ARDS are characterized by high-protein pulmonary edema and severe hypoxemic respiratory failure [1], [2], [3] and may result from many clinical insults, including sepsis and pneumonia. Despite improved understanding of the pathophysiology of ALI/ARDS, the mortality rate remains significant at 35–40% [2], [3], [4].

The key pathophysiologic feature of ALI/ARDS is injury to the pulmonary alveolar-capillary membrane [1], [2]. In sepsis, pulmonary microvascular endothelial cell (PMVEC) injury and barrier dysfunction results in the leak of protein-rich edema fluid and circulating neutrophils into the pulmonary interstitium and alveolar spaces [5], [6], [7]. PMVEC hyper-permeability during sepsis/ALI is the result of a complex interaction of PMVEC with many soluble factors, such as bacterial lipopolysaccharide (LPS) and endogenous pro-inflammatory cytokines (eg. tumour necrosis factor [TNF] α, interleukin [IL] 1β), as well as inflammatory cells, including circulating neutrophils and pulmonary-resident alveolar macrophages [6], [8], [9], [10], [11].

Alveolo-capillary PMVEC are normally closely apposed to alveolar epithelial cells (AEC), and both cells together regulate gas-exchange across the alveolo-capillary membrane. AEC also play a key role in keeping alveoli relatively “dry”, as they form a very tight permeability barrier [12]. AEC also continuously remove liquid from the alveolar space through the cationic and water channels located at the AEC apical surface, transporting fluid to the interstitial space for subsequent lymphatic removal [13]. AEC dysfunction, resulting in impairment of their barrier and water clearance functions, has been described in ARDS patients and is associated with worse outcome. AEC may also contribute to inflammatory events in ALI/ARDS, as they are an important source of cytokines (eg. TNFα, IL1β, IL6) and chemokines (eg.monocyte chemotactic protein [MCP] 1, IL8) under inflammatory conditions [14], [15], [16], and also promote intra-alveolar coagulation [17]. However, the potential biological importance of epithelial-endothelial interactions at the alveolo-capillary barrier, and specifically the effect of AEC presence on PMVEC permeability is not known, especially under septic conditions.

Thus, we hypothesized that AEC can modulate human PMVEC barrier function. Specifically, we assessed whether the presence of A549 cells, a human AEC cell line, or A549-derived soluble products can modulate human PMVEC barrier function under septic conditions in vitro. PMVEC isolated from human lung tissue were cultured in the presence or absence of A549 cells or primary human AEC during stimulation with LPS, cytomix (an equimolar mixture of clinically-relevant human cytokines TNFα, IL1β, and interferon [IFN] γ), or plasma isolated from patients with severe sepsis. In addition, we assessed the effects on septic PMVEC hyper-permeability of treatment with cell culture medium “conditioned” by prior incubation with A549 cells. The presence of A549 cells or A549 cell-conditioned medium (CM) attenuated PMVEC hyper-permeability under all septic conditions, through release of soluble factor(s), which were heat-stable and at least partly lipid in nature. In addition, A549 cell presence and A549-CM reduced septic neutrophil-dependent PMVEC hyper-permeability and attenuated trans-PMVEC neutrophil migration.

## Methods

### Ethics Approval

All procedures were approved by Western University’s Health Sciences Research Ethics Board. Informed written consent was obtained from healthy blood donors, as well as from septic patients or their surrogate decision-makers. Discarded lung tissue samples were obtained from the pathology department following removal of all clinical identifiers, and as such, the institutional review board waived the need for written informed consent from lung tissue donors.

### Human Pulmonary Microvascular Endothelial Cell (PMVEC) Isolation and Culture

PMVEC were isolated from human lung tissue, following resectional surgery for localized lung cancer, as previously reported [10].

### Human Alveolar Epithelial Cell (AEC) Isolation & Culture

Two different human AEC-type cells were used in these experiments. A549 cells, a type II AEC-like cell line, were cultured as per supplier`s guidelines (ATCC, Rockville, MD), and used for the majority of studies.

In certain key experiments, the effects of A549 cells were confirmed by limited studies using primary human type II AEC, which were isolated using a previously described method [18], [19]. Briefly, human peripheral lung tissue was finely minced, washed with PBS, filtered (100 µm strainer), and serially digested with dispase and then elastase/trypsin (Sigma). The digested tissue was filtered serially through sterile gauze, 100 µm, and 40 µm strainers, the filtrate washed with PBS, and incubated for 3-hours in 10% FBS/DMEM at 37°C with 5% CO_2_. The non-adherent cells were collected and centrifuged on a Percoll discontinuous gradient (1.089 g/ml and 1.040 g/ml, Sigma) at 300 g for 20 min. The cells at the interfacial layer were collected, washed twice with PBS, and 2×10^6^ cells seeded in each well of a 24-well plate and cultured in 10% FBS/DMEM. The next day, dead cells were removed, and attached cells permitted to grow to confluence over 4–5 days. >90% of cultured cells were AEC type II (positive staining with rabbit polyclonal anti-human surfactant protein-C antibody; Millipore, Billerica, MA), and were used for experiments within 6 days.

### Human PMVEC/A549 Cell Co-culture

In order to mimic the close apposition of PMVEC and AEC in the normal human lung, a bilayer of PMVEC/A549 cells was prepared as previously reported [19]. Cell-culture inserts with 3 µm pores (Costar, Cambridge, MA) were inverted and the basal undersurface of the membrane coated with 1% gelatin. 3×10^4^ A549 cells in 50 µL Endothelial Growth Medium (EGM; Walkerville, MD) were seeded on the basal undersurface of the insert and cultured for 3-hours at 37°C in 95% air/5% CO_2_. After incubation, the non-adherent cells were removed by aspiration. The inserts were then flipped upright in a 24-well plate, and incubated with 600 µL of EGM medium in the lower chamber. 10^5^ human PMVEC in 200 µL medium were seeded on the apical (upper) surface of the inserts. Every 3 days the medium was changed, and the experiments were carried out on day 5.

In some experiments, PMVEC/A549 cells were also co-cultured but were physically separated by a distance of 1 mm by culturing A549 cells in the lower chamber below PMVEC monolayers on cell-culture inserts. For these experiments, 10^5^ A549 cells were seeded in each lower chamber with 10% DMEM and cultured for 3 days to reach confluence. For PMVEC/A549 cell co-culture experiments, PMVEC monolayer-inserts were placed in the lower chambers containing A549 cells, along with 200 µL and 600 µL fresh EGM in upper and lower chambers, respectively.

### Isolation of Human Septic vs Control Plasma

Patients with severe sepsis were identified according to standard criteria [20], and recruited without consideration of their age, gender, or infection category. Isolated septic plasma was either used fresh or stored at −20°C. Control plasma was isolated from healthy volunteers.

### Preparation of A549 Cell-conditioned Medium

Confluent A549 cells were cultured in 24-well plates in 600 µL of fresh 10% EGM medium for 8-hours in the presence or absence of cytomix (10 ng/mL). This medium “conditioned” by A549 cells (A549-CM) medium was aspirated, centrifuged for 5 min at 1000×g at 4°C, and the supernatant kept at −20°C until use. Two control cell-CM were similarly prepared from: (1) cultures of human PMVEC, and (2) PMVEC/A549 cell co-cultures.

In pilot studies, the potential nature of a soluble A549 cell-derived factor in A549-CM was explored in three ways. First, A549-, PMVEC- and PMVEC/A549-CM were frozen at −80°C for later analysis of a panel of inflammatory cytokines, chemokines, and growth factors using multiplexed immunoassay kits, according to manufacturers’ instructions (Bio-Rad Laboratories Inc.,Hercules, CA). A Bio-Plex 200 readout system was used (Bio-Rad), which utilizes Luminex® xMAP fluorescent bead-based technology (Luminex Corporation, Austin,TX). Cytokine levels (pg/mL) were automatically calculated from standard curves using Bio-Plex Manager software (v. 4.1.1, Bio-Rad).

Second, non-cytomix-treated A549-CM was heated to 100°C for 20-min in order to denature peptides, and the effect of this heated A549-CM on septic PMVEC then assessed. Finally, non-cytomix-treated A549-CM was subjected to lipid extraction as previously described [21], [22]. Briefly, 25 mL of A549-CM or EGM was added to 94 mL chloroform/methanol/12N HCl (vol/vol/vol: 2/4/0.1). The solution was mixed thoroughly, followed by the addition of 37 mL of chloroform and further mixing. Subsequently, 25 mL of H_2_O was added and the solution was mixed via 5-min of shaking, and centrifuged at 1750×g for 10-min. The upper aqueous (water-methanol) phase was removed via suction, and the lower chloroform phase was dried under N_2_ gas. The dissolved lipid extract was dried and stored under N_2_ gas until further use. For experiments, the extracted lipids were dissolved in 250 µL methanol and sonicated, after which 20 µL of lipid extract from A549-CM or EGM was added to 1 mL of EGM. Additionally, 20 µL of methanol was similarly diluted in the control medium, to a final concentration of 2% for lipid extract experiments.

### Experimental Protocols

#### Trans-PMVEC albumin permeability studies

Human PMVEC monolayers in cell-culture inserts were stimulated for 8-hours with cell-culture medium containing LPS (E. Coli, 055:B5; Sigma), cytomix (an equimolar mixture of sepsis-relevant human cytokines TNFα, IL1β, and IFNγ; Sigma), or plasma from septic patients vs healthy control subjects vs appropriate control media. For LPS and cytomix studies, the appropriate control treatment was PBS-treated culture medium. For human plasma studies, the relevant control treatment chosen was 20% fetal bovine serum (FBS)-treated culture medium.

For LPS and cytomix experiments, the respective septic stimulation was added to both upper and lower chambers. In pilot studies, stimulation in only upper or lower chambers was less effective than stimulation in both chambers. For human plasma studies, plasma or the control FBS were only added to the apical aspect of PMVEC (upper chamber), because apical plasma exposure was thought to be more physiologic and because of limited supplies of individual patient plasma for the number of required studies. Moreover, in pilot studies, septic plasma stimulation of PMVEC was more effective from the upper vs lower chamber, and equally effective to septic plasma stimulation in both upper/lower chambers (data not shown).

The permeability of PMVEC monolayers or PMVEC/A549 cell bilayers was assessed by measuring the flux of Evans blue (EB) dye-labeled bovine serum albumin, as we previously reported [9], [10]. After 7-hours of the 8-hours septic stimulation, 150 µl of a 4% solution of albumin were added to the lower chamber and 50 µl of a 0.67 mg/ml EB-albumin solution were added to the upper chamber. The trans-PMVEC flux of EB-albumin into the lower chamber over exactly 1-hour was quantified by measuring the absorbance of the medium in the lower chamber at 620 nm, and comparing values to a standard curve.

#### Polymorphonuclear neutrophil (PMN) dependent trans-PMVEC protein leak, migration and adhesion

For all PMN-PMVEC experiments, PMNs were isolated from normal human blood donors using Lymphocyte Separation Medium (ICN Biomedicals Inc., Aurora, OH), as described previously [23]. For quantification of PMN-PMVEC adhesion and trans-PMVEC PMN migration, isolated PMNs were labeled with calcein-AM (excitation 494 nm, emission 517 nm; Invitrogen, Burlington, Ontario, Canada).

PMN-dependent PMVEC hyper-permeability and trans-PMVEC PMN migration were assessed in cell-culture inserts. Confluent PMVEC monolayers and PMNs were separately stimulated with cytomix (0.03 and 0.3 ng/mL) for 3-hours. 5×10^5^ PMN were then added to the apical aspect of PMVEC monolayers for 1-hour with cytomix (0.03 and 0.3 ng/mL) stimulation. Following PMN-PMVEC co-culture, EB-albumin was added to the upper chamber of inserts, and 1-hour later, the medium from the lower chamber of the inserts (containing EB-albumin and migrated PMN) was collected and centrifuged for 5-min at 500 g. Trans-PMVEC EB-albumin leak was measured spectrophotometrically in the supernatant. The cell pellets were resuspended, and calcein-AM-labeled PMN quantified by fluorescence spectrophotometry (Victor3 fluorescence plate reader, PerkinElmer, Boston, MA) against a standard curve of number of calcein-AM-labelled PMN.

PMN-PMVEC adhesion was assessed in 24 well plates. Confluent PMVEC monolayers and PMNs were separately stimulated with cytomix (0.03 and 0.3 ng/mL) for 3-hours, and then 5×10^5^ PMNs were added to the apical aspect of PMVEC monolayers for 1-hour under cytomix (0.03 and 0.3 ng/mL) stimulation. Non-adherent PMNs were removed by washing the monolayers twice with PBS. The remaining cells in the wells were collected with a cell scraper and the fluorescence intensity of the cell suspension was measured. PMN-PMVEC adhesion rates were calculated according to a standard curve as above.

### Statistical Analysis

Data represent the mean±SEM of at least 3 independent experiments performed in at least duplicate. Data were analyzed using one-way ANOVA, with post hoc testing where indicated using Dunnett’s multiple comparison test. P<0.05 was accepted as significant.

## Results

### Effects of A549 Cell Presence on Septic PMVEC Hyper-Permeability

**Figure 1:**
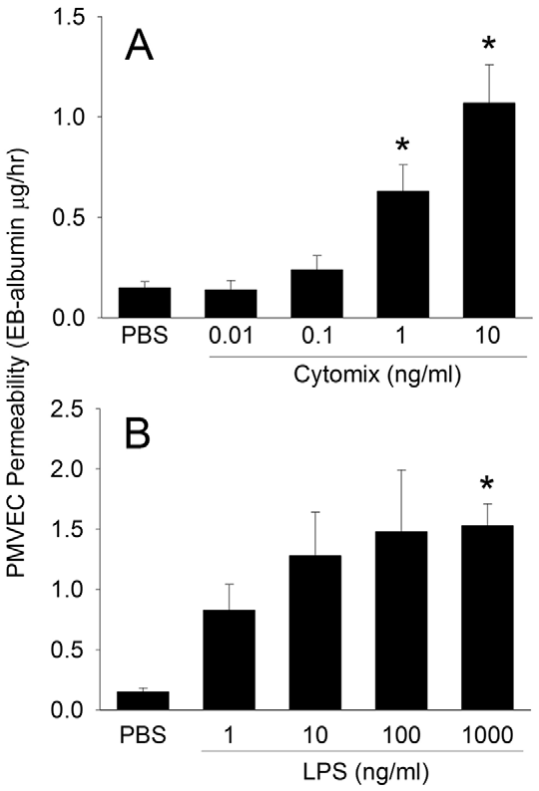
Dose-dependent effects of cytomix and lipopolysaccharide (LPS) on human pulmonary microvascular endothelial cell (PMVEC) permeability. PMVEC seeded on cell-culture inserts for 5–6 days formed a monolayer, which was subsequently stimulated with either cytomix (A) or LPS (B) vs PBS for 8-hours, and the permeability determined by measurement of trans-PMVEC leak of Evans blue (EB) dye-labeled albumin over the final 1-hour. The dose-response effects of cytomix and LPS were both significant (P<0.01) by ANOVA; *: P<0.05 vs. PBS/non-stimulated groups.

Septic treatment of isolated human PMVEC with either cytomix or LPS resulted in a significant and dose-dependent increase in human PMVEC permeability to EB-labeled albumin (Fig. 1). This “septic” PMVEC hyper-permeability did not appear to be due simply to PMVEC death, as PMVEC survival was 97±2% and 98±1% at maximal doses of cytomix and LPS, respectively, no different than 99±1% for PBS (p = ns for both).

**Figure 2:**
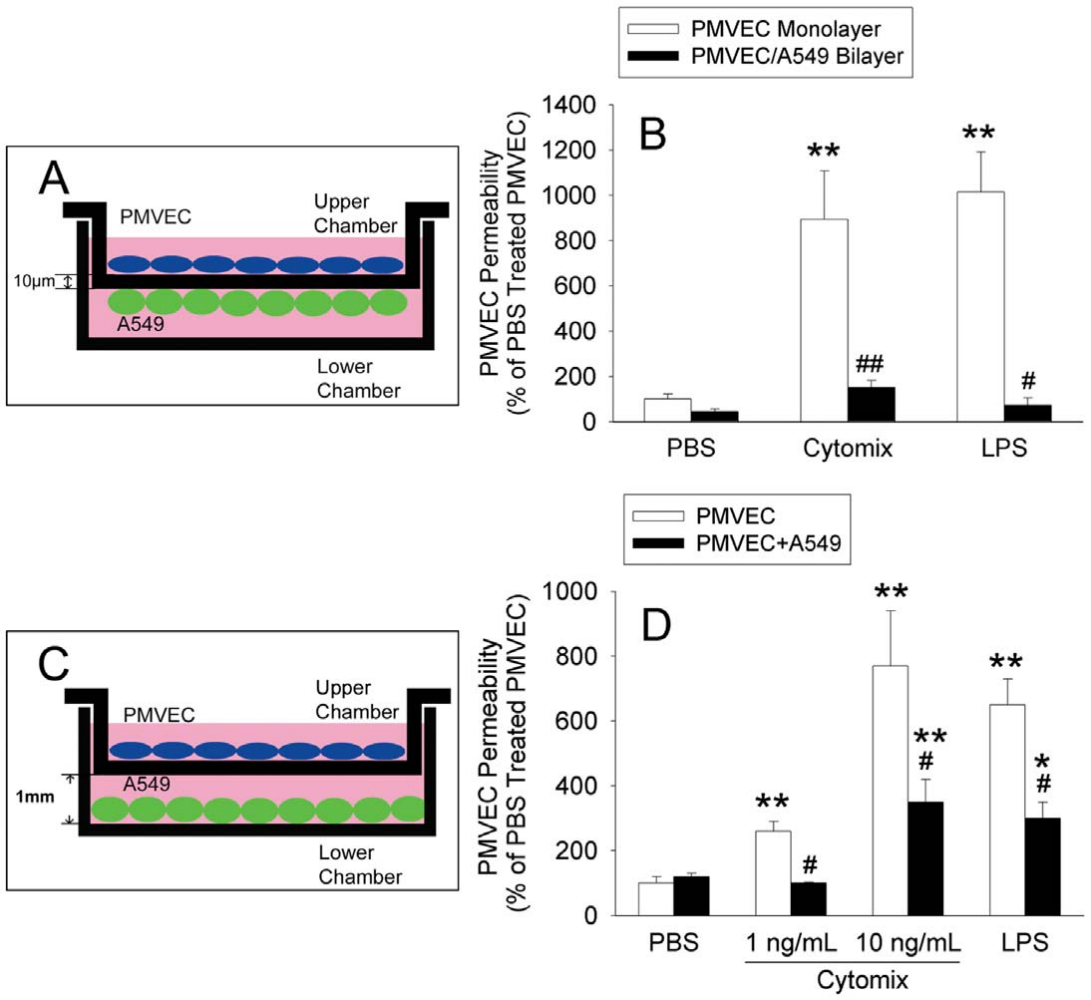
Effect of A549 cells in the bottom chamber on septic trans-PMVEC hyper-permeability. (A) PMVEC monolayers on the apical aspect of inserts were co-cultured with A549 cells on the under-surface of the insert membrane, forming a bilayer. (B) PMVEC were stimulated with cytomix (10 ng/mL) or LPS (1 µg/mL) for 8-hours to assess the effects of the presence (filled bars) or absence (open bars) of adjacent A549 cells on trans-PMVEC EB-albumin leak. (C) PMVEC on inserts were also co-cultured with A549 cells on the bottom surface of the lower chamber, at 1 mm distance from the insert membrane. (D) The effects of the presence (filled bars) or absence (open bars) of remote A549 cells on trans-PMVEC EB-albumin leak following cytomix (1 or 10 ng/mL) or LPS (1 µg/mL) stimulation for 8-hours were assessed. P<0.001 by ANOVA. *: P<0.05 or **: *P*<0.01 vs. respective PBS; #: *P*<0.05 for the presence of A549 cell vs. PMVEC alone.

We next assessed the effect of the presence of A549 cells in co-culture with PMVEC on this septic PMVEC hyper-permeability in 2 ways. First, a bilayer of PMVEC/A549 cells was created in vitro as a model of the close apposition of PMVEC and AEC, separated only by a thin basement membrane in the normal human alveolo-capillary membrane (see schema, Fig. 2A). In the PMVEC/A549 cell bilayer model, septic PMVEC hyper-permeability following cytomix or LPS stimulation was completely eliminated (Fig. 2B). In a second approach, the potential effects of A549 cells on septic PMVEC hyper-permeability were assessed in a PMVEC/A549 cell non-contact co-culture model, by culturing human PMVEC on cell-culture inserts in the presence vs absence of A549 cells in the lower chamber (see schema, Fig. 2C). Even at this “remote” location, at 1 mm distance from PMVEC, A549 cells significantly reduced septic PMVEC hyper-permeability by 100±3% and 64±9% for low-dose (0.1 ng/mL) and high-dose (10 ng/mL) cytomix, respectively (Fig. 2D). Similarly, A549 cells attenuated LPS-induced trans-PMVEC EB-albumin leak by 61±4%. In control studies, the presence of extra PMVEC, rather than A549 cells, in the lower chamber, had no effect on septic PMVEC hyper-permeability (data not shown).

**Figure 3:**
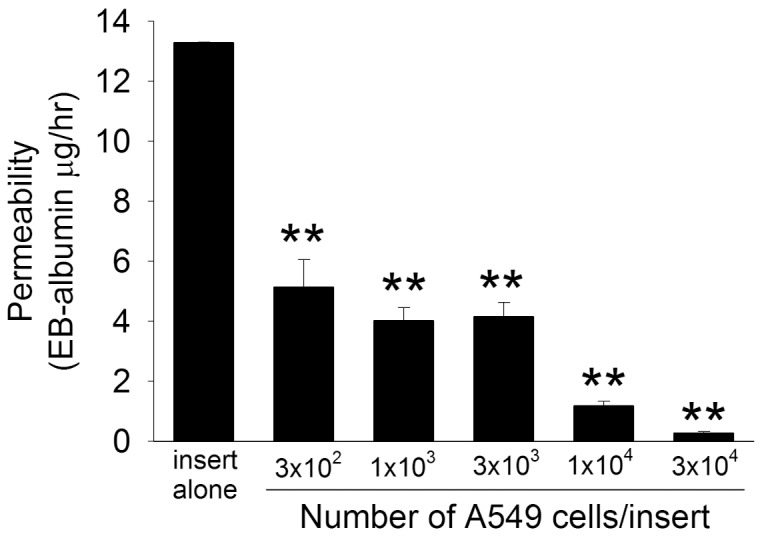
The effect of cell number on permeability of isolated alveolar epithelial cells (A549). A549 cells at varying density were seeded onto cell-culture inserts, incubated for 72-hours, and the trans-A549 cell permeability to EB-labeled albumin assessed over 1-hour incubation. P<0.01 by ANOVA; **, P<0.01 vs insert alone.

Although PMVEC barrier properties are important in normal alveolo-capillary function, AEC also form a very tight permeability barrier [12]. Thus, in the PMVEC/A549 cell bilayer model, we were concerned whether the apparent reduction in septic PMVEC hyper-permeability in the presence of adjacent A549 cells was simply due to A549 cells creating a physical barrier. Indeed, when we assessed the permeability of isolated A549 cells by themselves, in the absence of PMVEC, they dose-dependently and dramatically decreased EB-albumin leak (Fig. 3). As such, it was clear that any potential modulatory effects of A549 cells on PMVEC permeability could not be assessed via this PMVEC/A549 cell bilayer model. Based on the inability to directly assess PMVEC permeability in the PMVEC/A549 cell bilayer model, all subsequent studies were completed using the PMVEC/A549 cell non-contact co-culture model.

**Figure 4:**
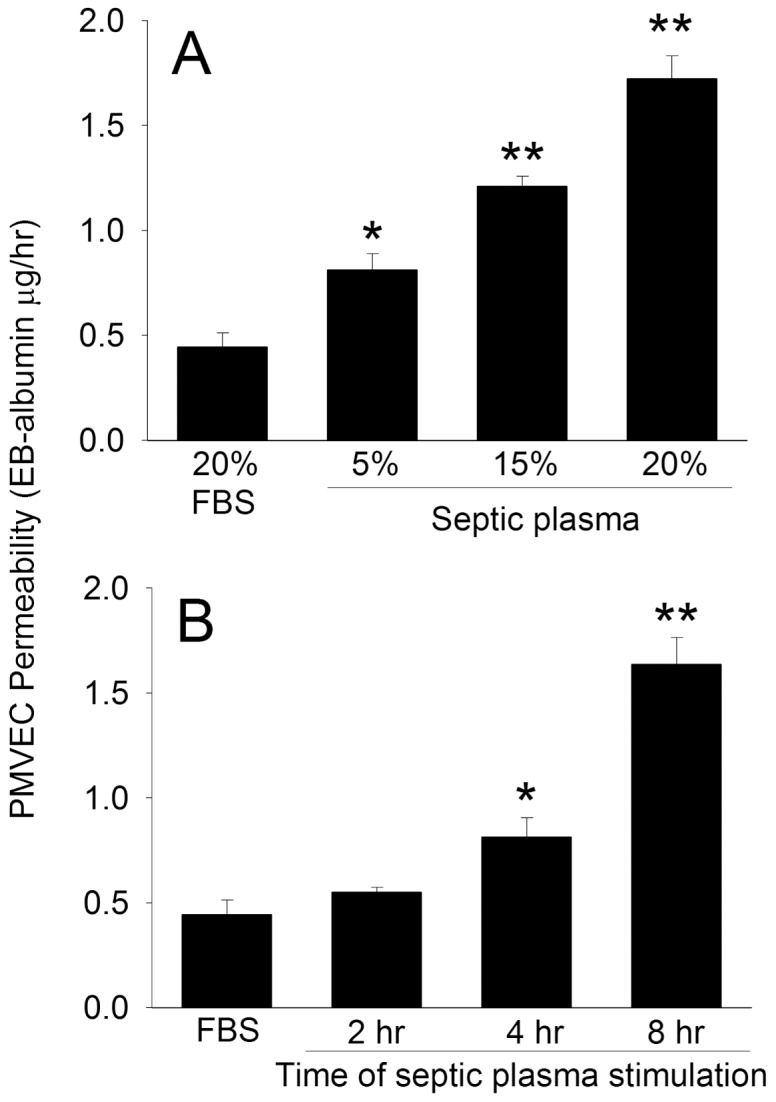
Characterization of the dose-response and time course of septic plasma treatment on trans-PMVEC EB-albumin leak. (A) PMVEC on the apical aspect of inserts were cultured for 8-hours with varying concentrations of plasma isolated from 3 different patients with sepsis, and trans-PMVEC EB-albumin leak measured over the final 1-hour. (B) PMVEC on the apical aspect of inserts were treated with 20% plasma from 3 different septic patients for a total of 2-, 4-, or 8-hours, and trans-PMVEC EB-albumin leak measured over the final 1-hour. P<0.05 by ANOVA for both A and B; *: P<0.05 and **: P<0.01 vs FBS control.

The clinical relevance of this putative A549 cell protective effect against septic PMVEC hyper-permeability was assessed by treating human PMVEC monolayers with plasma isolated from septic human patients. In pilot experiments, apical (upper chamber) septic plasma treatment induced PMVEC hyper-permeability to EB-albumin in a dose-dependent manner (Fig. 4A), beginning at 4-hours after stimulation, and peaking at 8-hours (Fig. 4B). Thus, 20% plasma and 8-hours stimulation time were used for all subsequent human plasma experiments.

**Figure 5:**
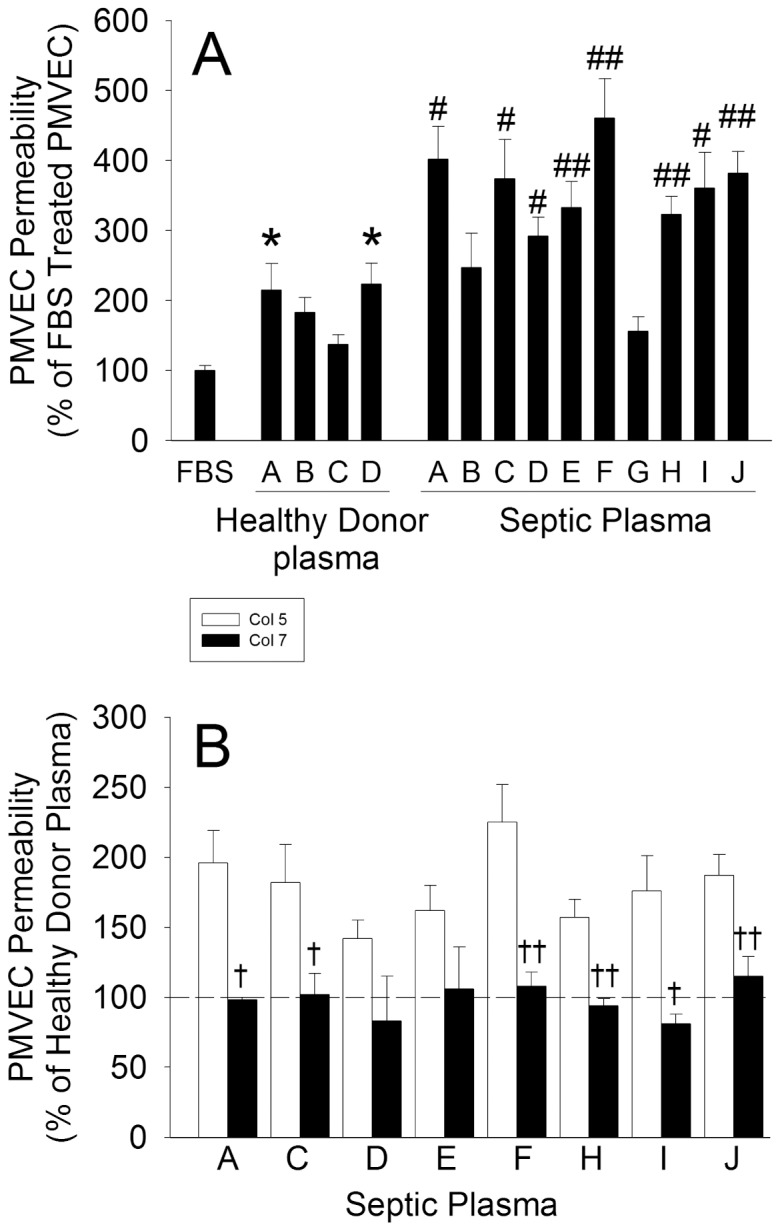
Effect of A549 cells (bottom chamber) on trans-PMVEC hyper-permeability following septic plasma treatment. (A) PMVEC on the apical aspect of inserts were cultured for 8-hours with 20% FBS control, 20% plasma from 4 different healthy donor control subjects, or 20% plasma from 10 different patients with sepsis, and trans-PMVEC EB-albumin leak measured over the final 1-hour. *: *P*<0.05 vs FBS (note: all septic plasma bars were significantly different from FBS control, but this is not indicated in the figure to avoid clutter); #: *P*<0.05 and ##: P<0.01 vs average of healthy donor control plasma. (B) PMVEC on the apical aspect of inserts were treated with 20% plasma from septic patients (n = 8) for 8-hours in the presence (filled bars) or absence (open bars) of A549 cells cultured on the bottom surface of the lower chamber. The dotted line represents the average level of EB-albumin leak induced by healthy donor control plasma in panel A. P<0.05 by ANOVA. †: *P*<0.05 and ††: P<0.01 for the presence of A549 cells vs. PMVEC alone.

Human plasma isolated from healthy blood donors increased trans-PMVEC EB-albumin leak by 190±20% vs control FBS (Fig. 5A). Plasma from 8 of 10 septic human subjects induced marked septic PMVEC hyper-permeability, increasing trans-PMVEC EB-albumin leak by 366±19% of control FBS, which was greater than the effect of healthy-donor control plasma (p<0.05). Moreover, the presence of A549 cells in the lower chamber almost completely attenuated human septic plasma-induced PMVEC hyper-permeability, back to the level of permeability seen with healthy-donor control plasma (Fig. 5B).

### Effects of A549 Cell-Conditioned Medium (CM)

**Figure 6:**
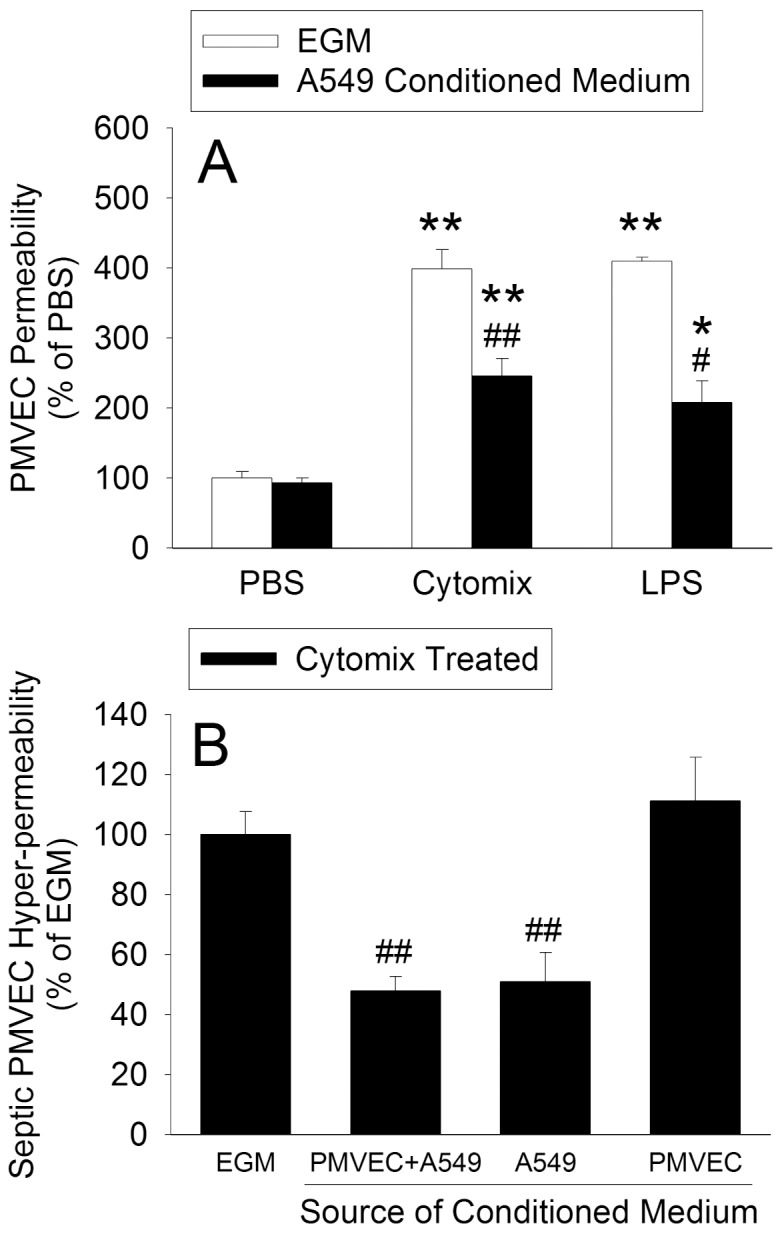
Effects of incubation of PMVEC in A549 cell-conditioned medium (CM) on trans-PMVEC hyper-permeability under septic conditions. (A) A549 cell-conditioned medium (CM) was generated by culturing A549 cells alone in Endothelial Growth Medium (EGM) for 8-hours. PMVEC were subsequently cultured in EGM or in this A549-CM during stimulation with cytomix (10 ng/mL) or LPS (1 µg/mL) for 8-hours. (B) Conditioned medium was generated from PMVEC+A549 cells in co-culture, A549 cells alone, or PMVEC alone. PMVEC were subsequently cultured in EGM or in the various conditioned media during stimulation with cytomix(10 ng/mL) for 8-hours. The data are expressed as a percentage of cytomix-stimulated septic trans-PMVEC EB-albumin hyper-permeability. P<0.01 by ANOVA for A and B. *: P<0.05 and **: P<0.01 vs respective PBS; #: *P*<0.05 and ##: P<0.01 vs respective EGM-treated.

We then explored the putative presence of soluble factors produced by A549 cells in the protective effect against septic PMVEC hyper-permeability. Unstimulated A549 cells were cultured for 8-hours in normal cell-culture medium, and this A549-CM collected, and subsequently used to culture PMVEC during treatment with cytomix or LPS. A549-CM significantly protected against septic (both cytomix and LPS) PMVEC hyper-permeability (Fig. 6). Moreover, CM isolated from co-cultured PMVEC/A549 cells, but not from PMVEC alone, had a similar protective effect against septic PMVEC hyper-permeability. Thus, the protective effect against septic PMVEC hyper-permeability was specifically dependent on the presence of A549 cells.

**Figure 7:**
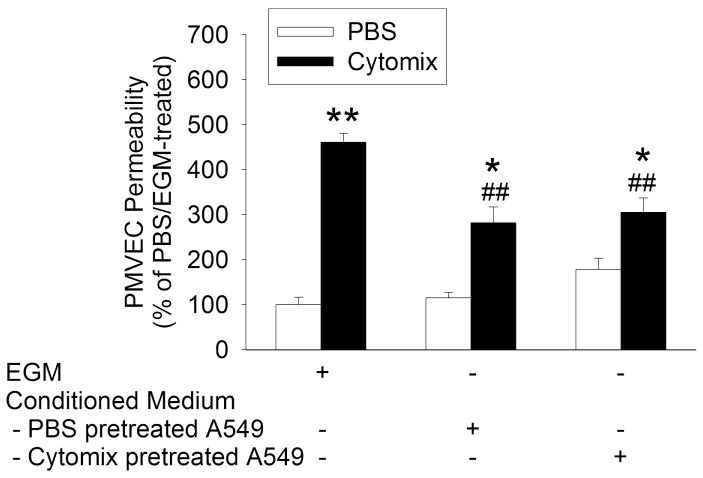
Effect of cytomix stimulation of A549 cells on the protective effect of A549 cell-conditioned medium (CM) against cytomix-stimulated septic PMVEC hyper-permeability. Conditioned medium was obtained by prior incubation of A549 cells in EGM with or without concurrent treatment with cytomix (10 ng/mL). PMVEC were subsequently cultured in EGM or in A549-CM (PBS vs cytomix pre-treated) during stimulation with cytomix (10 ng/mL) for 8-hours. P<0.01 by ANOVA. *: P<0.05 and **: P<0.01 vs respective PBS; ##: P<0.01 vs Cytomix/EGM-treated.

In order to assess the effects of “septic” stimulation on A549 cells and the protective mechanism against septic PMVEC hyper-permeability, A549 cells were pre-stimulated with cytomix (10 ng/mL) for 8-hours prior to collection of A549-CM. Cytomix pre-treatment of A549 cells did not affect the protective effects of A549-CM against septic PMVEC hyper-permeability (Fig. 7).

### Effects of Primary Human Type II AEC Presence on Septic PMVEC Hyper-Permeability

**Figure 8:**
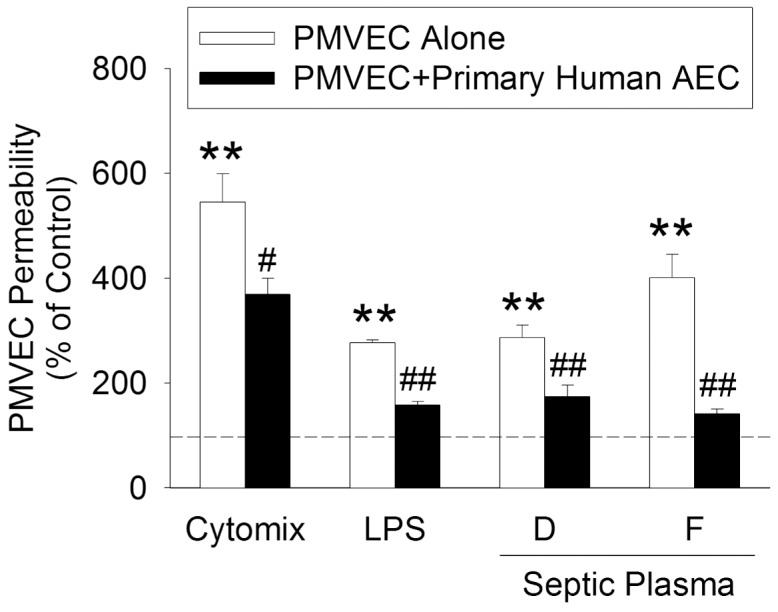
Effect of primary human alveolar epithelial cells in the bottom chamber on septic PMVEC hyper-permeability. PMVEC on the apical aspect of inserts were stimulated with cytomix (10 ng/mL), LPS (1 µg/mL), or 20% plasma from septic patients (n = 2) for 8-hours in the presence (filled bars) or absence (open bars) of primary human alveolar epithelial cells (AEC) cultured in the bottom chamber. The dotted line represents the appropriate control signal for each stimulus (PBS for cytomix and LPS; FBS for plasma). *: P<0.05 and **: P<0.01 vs respective control; #: P<0.05 and ##: P<0.01 vs PMVEC alone.

The putative A549 cell-mediated protective effect on septic human PMVEC was confirmed in a small number of selected experiments by isolating and studying primary human type II AEC from three different human subjects. These primary human AEC were cultured in the lower chamber below human PMVEC, in a PMVEC/AEC non-contact co-culture model, in the presence of cytomix, LPS, or septic plasma. Similar to the protective effect of A549 cell presence in PMVEC/A549 co-culture in the studies described above, the presence of primary human type-II AEC significantly attenuated PMVEC septic hyper-permeability induced by cytomix, LPS, or septic plasma by 39±4%, 69±4%, and 73±8%, respectively (Fig. 8).

### Effects of A549 Cells on Septic Neutrophil-PMVEC Interactions

**Figure 9:**
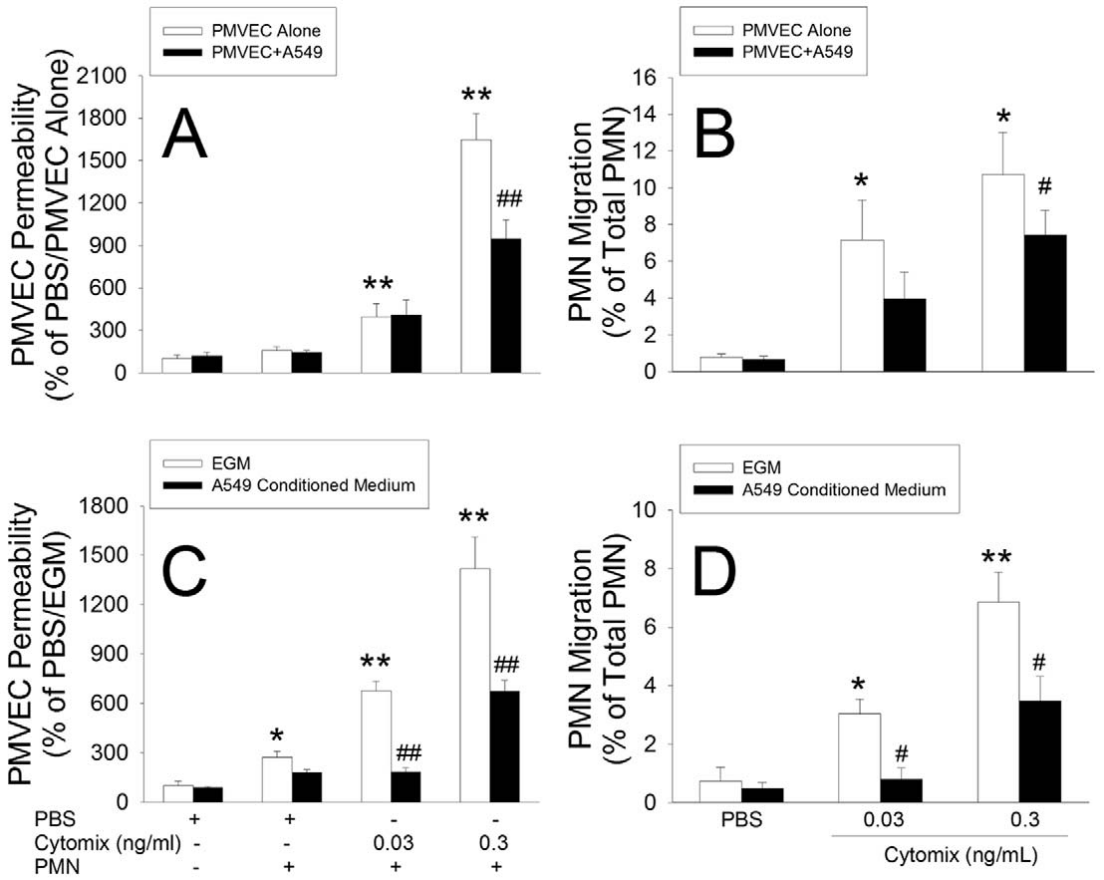
Effect of A549 cells and A549 cell-conditioned medium (CM) on septic PMN-dependent PMVEC permeability and trans-PMVEC PMN migration. Panels A,B: In cytomix-stimulated PMN/PMVEC co-cultures, PMVEC permeability and PMN migration were measured in the presence vs absence of A549 cells (non-contact co-culture model). Septic PMN-dependent PMVEC hyper-permeability and trans-PMVEC PMN migration were inhibited in the presence of A549 cells. Panels C,D: Similarly, A549-CM inhibited both septic PMN-dependent PMVEC hyper-permeability and trans-PMVEC PMN migration. *: P<0.05 and **: P<0.01 vs respective control; #: P<0.05 and ##: P<0.01 vs PMVEC alone (panels A,B) or EGM-treated (panels C,D).

The effects of A549 cell presence on septic PMVEC hyper-permeability in PMVEC/A549 cell co-culture were assessed in a more clinically relevant situation of neutrophil presence. Human neutrophil presence greatly enhanced septic PMVEC hyper-permeability following treatment with cytomix (0.03 and 0.3 ng/mL; Fig. 9A). The presence of A549 cells significantly reduced neutrophil-dependent septic PMVEC hyper-permeability. In addition, septic stimulation was associated with increased trans-PMVEC neutrophil migration, which was also markedly reduced by A549 cell presence (Fig. 9B).

Next, we assessed the effects of A549-CM, rather than the presence of A549 cells, in neutrophil-PMVEC co-cultures. Similar to the inhibitory effect of A549 cell presence, treatment with A549-CM significantly reduced both neutrophil-dependent septic PMVEC hyper-permeability (Fig. 9C) as well as trans-PMVEC neutrophil migration (Fig. 9D).

In neutrophil-PMVEC co-culture, neutrophil adhesion to PMVEC was also increased under septic conditions (Table 1). However, in contrast to the inhibitory effects of A549-CM on trans-PMVEC neutrophil migration, A549-CM treatment of neutrophil-PMVEC co-cultures had no effect on neutrophil-PMVEC adhesion.

**Table 1:** Cytomix-induced PMN adhesion to PMVEC (% of total PMN).

Medium	PBS	Cyto 0.03 ng/mL	Cyto 0.3 ng/mL
EGM	0.46±0.03	3.8±0.7**	10.2±2.3**
A549-CM	0.39±0.06	3.3±0.7**	8.6±1.8**

** P<0.01 vs PBS. n = 8.

### Exploratory Studies on the A549 Cell-Derived Soluble Protective Factor against Septic PMVEC Hyper-Permeability

In exploratory studies, the production by A549 cells of an array of sepsis-relevant soluble cytokines, chemokines, and growth factors was measured by multiplex ELISA (Table 2). In isolated, unstimulated (PBS-treated) A549 cells in culture, there was release of low levels of IL8 and MCP1 over 8-hours, but no production of any other measured factors. In contrast, following cytomix stimulation, A549 cells released significant levels of IL6 and IL8, macrophage inflammatory protein (MIP1β), interferon gamma-induced protein 10 (IP10), MCP1, and granulocyte colony-stimulating factor (GCSF) over 8-hours. In contrast, we did not detect synthesis of any IL9, IL15, IL17, or platelet-derived growth factor (PDGF) from cytomix-treated A549 cells (data not shown). PMVEC alone produced much lesser amounts of IL8, IP10, and GCSF than A549 cells, and there was no appreciable production of MIP1β. In PMVEC/A549 cell co-culture, there was a significantly greater, synergistic increase in the culture medium levels of IL6, IL8, and GCSF, but a lesser production of IP10 than in isolated A549 cells alone.

**Table 2:** Levels of cytokines or chemokines in medium (pg/ml).

			A549			PMVEC/A549			PMVEC		
	Time (hr)		PBS	Cytomix		PBS	Cytomix		PBS		Cytomix
IL-8		0	4±0		7±1	7±0		10±0	―	3±0	
		2	48±7*		1992±99* †	151±5* ^#^ †		19679±3277* ^#^ †	49±8*	152±33*	
		4	49±7*		21973±2845* †	190±24* ^#^ †		44981±8* ^#^ †	106±13*	2849±336*	
		8	73±5*		62481±280* †	214±17* ^#^ †		129671±82* ^#^ †	68±7*	26298±*	
MIP-1±		0	―		2±0	―		2±0	―	―	
		2	―		35±3* †	―		47±8* †	―	―	
		4	―		676±65* †	―		1280±187* †	―	―	
		8	―		1104±114* †	―		1272±211* †	―	―	
IP-10		0	―		12±2	―		11±2	―	13±2	
		2	―		936±56* †	―		91±12* ^#^ †	―	15±3	
		4	―		20157±2184* †	―		1851±138* ^#^ †	―	468±84*	
		8	―		39913±2845* †	―		6819±522* ^#^ †	―	1519±497*	
MCP-1		0	3±1		5±2	9±2		11±2	―	―	
		2	25±3*		414±16* †	91±12		309±25* †	92±25	138±32	
		4	27±4*		1898±258* †	96±16		1577±148* †	115±31*	507±45*	
		8	33±3* †		2054±337* †	132±21		1904±286*	95±2*	1375±94*	
IL-6		0	―		5±0	―		5±0	―	4±0	
		2	―		58±3* †	―		199±23* ^#^ †	―	6±1	
		4	―		111±4* †	―		429±35* ^#^ †	―	50±10*	
		8	―		193±2*	―		682±95* ^#^ †	―	256±17*	
G-CSF		0	―		10±1	―		9±1	―	9±0	
		2	―		43±4* †	―		45±2* †	―	11±1	
		4	―		705±41* †	―		2628±781* †	―	226±28*	
		8	―		2383±347* †	―		22205±6192* ^#^ †	―	978±85*	

* P<0.05 vs respective time 0;

# P<0.05 vs A549 alone at the same time point;

† P<0.05 vs PMVEC alone at the same time point;

―: not detected.

**Figure 10:**
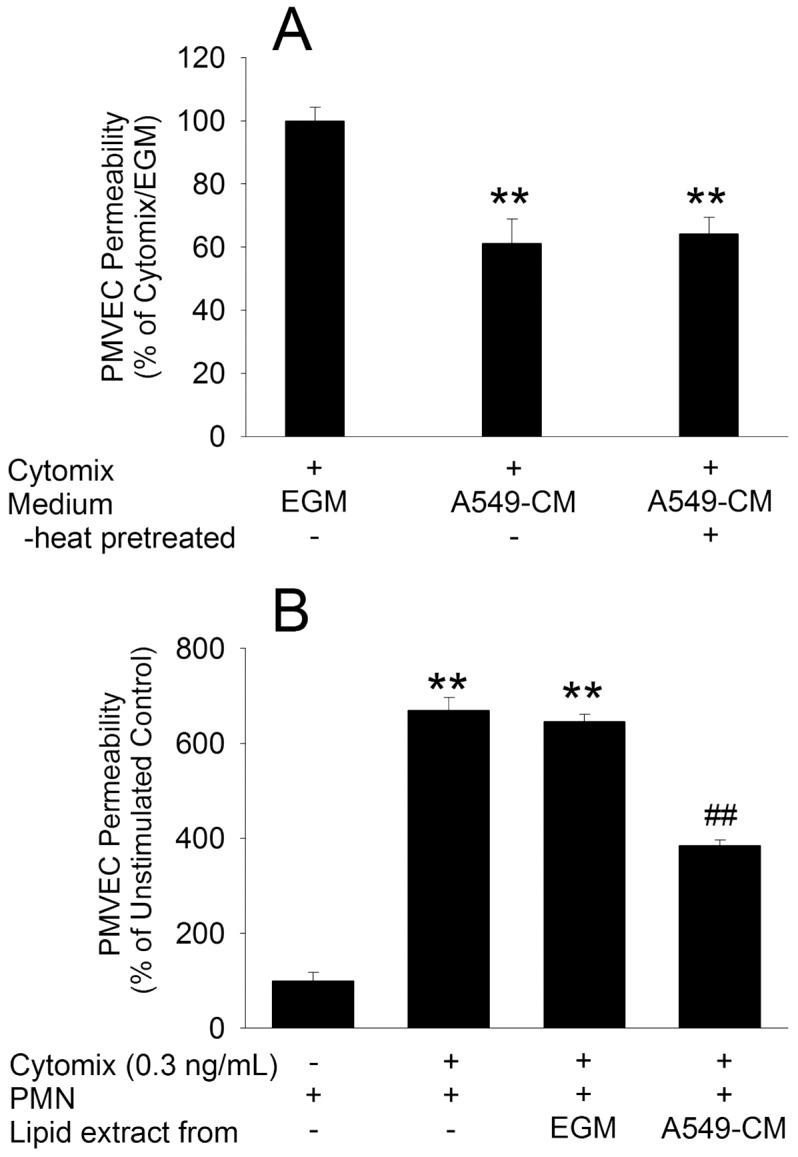
Pilot studies on nature of the A549 cell-derived soluble protective mediator(s) in A549 cell-conditioned medium (CM). Panel A: The protective effect of A549-CM against cytomix-stimulated septic PMVEC hyper-permeability was preserved following heat pre-treatment of the A549-CM. Panel B: The lipid extract from A549-CM, but not from EGM, protected against septic PMN-dependent PMVEC hyper-permeability. **: P<0.01 vs respective control; ##: P<0.01 vs cytomix-stimulated PMN-dependent PMVEC hyper-permeability.

Next, we assessed other possible properties of the soluble mediator(s) likely responsible for the protective effect of A549-CM on PMVEC hyper-permeability. Despite heat pre-treatment of the A549-CM, the protective effect against septic PMVEC hyper-permeability was preserved, suggesting that this heat-stable protective effect was not mediated by soluble protein(s) in the medium (Figure 10A). Moreover, the protective effect of A549-CM was transferable through a lipid extract from A549-CM, but not from EGM, protecting against septic neutrophil-dependent PMVEC hyper-permeability (Figure 10B). In control studies, unstimulated PMVEC permeability and septic PMVEC hyper-permeability, as well as the protective effect of A549-CM were not affected by the presence of 2% methanol in the lipid extraction process (data not shown).

## Discussion

In this study, septic treatment of human pulmonary microvascular endothelial cells in vitro with LPS or cytomix increased trans-PMVEC albumin leak. This septic PMVEC hyper-permeability was markedly attenuated by co-culture with A549 cells, a human alveolar epithelial cell line, both in a PMVEC/A549 cell bilayer and in a non-contact PMVEC/A549 cell co-culture model. Moreover, exposure of PMVEC to septic human plasma also induced significant PMVEC hyper-permeability, which was almost completely inhibited by co-culture with A549 cells. Primary human AEC also attenuated septic PMVEC hyper-permeability in response to LPS, cytomix, and septic plasma. Septic PMVEC hyper-permeability was also prevented by incubation of PMVEC in A549 cell-conditioned medium (CM). Furthermore, A549 cell presence and A549-CM protected against septic PMN-dependent PMVEC hyper-permeability and attenuated trans-PMVEC PMN migration. The putative A549 cell-derived soluble protective factor(s) were independent of septic stimulation of A549 cells, heat-stable, and transferable through a lipid extract of A549-CM.

The key pathophysiological mechanism of ALI/ARDS is hypoxaemic respiratory failure due to protein-rich alveolar edema, a result of increased permeability of the pulmonary alveolo-capillary microvascular barrier. Under normal conditions, the PMVEC barrier regulates the passage of fluid, macromolecules, and circulating cells from blood to the lung. However in ALI/ARDS, the effects of multiple inflammatory mediators and cells, as well as oxidative stress, increase PMVEC permeability and promote neutrophil-PMVEC adhesion and trans-PMVEC neutrophil migration into lung interstitial and alveolar spaces.

There is limited direct evidence of PMVEC injury in humans with ARDS. Plasma levels of three markers of EC activation/injury (Von Willebrand’s factor [VWF], intercellular adhesion molecule [ICAM] 1, and E-selectin) were elevated in 55 ARDS patients, with significantly higher levels in sepsis vs trauma patients [24]. Moreover, in an ARDSnet study, plasma VWF was elevated more than three-fold normal in ARDS patients, and higher levels of this marker of EC activation/injury correlated inversely with lower survival, as well as fewer ventilator-free and organ failure-free days [25]. Indirect evidence of PMVEC injury in ALI comes from animal models of sepsis/ALI [5], [7], [26], as well as study of human PMVEC in vitro, as in the present study. We have previously reported that human PMVEC septic albumin leak in vitro: (a) is enhanced by neutrophil presence and neutrophil-PMVEC adhesion, (b) is specifically neutrophil inducible nitric oxide synthase (iNOS)-dependent, and (c) appears to be mediated by peroxynitrite [9], [27].

The normal alveolar wall is comprised of many cells, including interstitial cells such as macrophages, but AEC and capillary endothelial cells comprise almost 50% of the alveolar wall cell population [28]. Two AEC types have been recognized to line alveoli: types I and II. Type I AEC are large, squamous cells which cover the majority of the alveolar surface and regulate gas exchange and solute/water diffusion [14]. Type II AEC are cuboidal cells that can proliferate and differentiate into type I AEC, repopulating the denuded alveolar lining in ARDS. Type II AEC also synthesize pulmonary surfactant, facilitate clearance of alveolar fluid and have an emerging role in innate immunity.

As we demonstrated, AEC constitute a very tight barrier, such that their permeability to albumin has been estimated at only 10% that of PMVEC [12]. As such, the PMVEC/AEC bilayer model was not an appropriate one to assess the effects of the presence of AEC on PMVEC septic hyper-permeability. Hence, we studied the effects of AEC (both A549 cell and primary human AEC) on septic PMVEC hyper-permeability through a non-contact, co-culture model. Even though AEC were co-cultured in the lower chamber of cell-culture inserts, at a 1 mm distance from PMVEC, we observed marked attenuation of PMVEC hyper-permeability under all septic conditions, including treatment with plasma from septic humans. These data suggest AEC-mediated protection against septic PMVEC hyper-permeability is mediated through release of some soluble factor(s), which was confirmed by similar protection observed following treatment of PMVEC with A549 cell-conditioned medium.

Our data strongly suggest a humoral mechanism, with release of some soluble factor(s) into the culture medium, which then acted in paracrine fashion on PMVEC to protect against septic hyper-permeability, but the nature of the soluble factor(s) remains unknown. We found that A549 cells produced high levels of a number of cytokines and growth factors in response to septic stimulation, such as IL6/8, MCP1, and GCSF [15], [29]. AEC can also secrete a number of other anti-inflammatory and anti-microbial factors such as lysozyme, beta defensins, cathelicidin, lipocalin-2, and various anti-oxidants [30] that could potentially protect PMVEC against septic hyper-permeability. However, none of the above factors are likely to be responsible for our observed A549 cell-mediated protection against septic PMVEC hyper-permeability for several reasons: (1) conditioned medium from both naive and cytomix-treated A549 cells had a similar protective effect; (2) the A549 cell-dependent protective factor(s) were heat-stable; and (3) the A549 cell-dependent protective effect was transferable through a lipid extract of A549 cell-conditioned medium.

Preliminary data from our exploratory studies on the nature of the A549 cell-derived soluble factor(s) suggest that lipid mediator(s) could be responsible for the protection against septic PMVEC hyper-permeability. Indeed, type II AEC can produce a number of lipid mediators, including surfactant [30], sphingosine-1-phosphate [31], epoxyeicosatrienoic acids (EETs) [32] and AEC-derived microparticles [33]. Clearly, a primary function of type II AEC is the production and release of pulmonary surfactant. Besides its crucial role in alveolar biomechanical homeostasis, surfactant consists of proteins and lipids that can modulate innate and adaptive immune systems. Surfactant-associated proteins A and D enhance clearance of microbes and regulate host inflammatory responses through interactions with multiple host cell types, including T cells, dendritic cells and eosinophils [34], [35]. The potential anti-inflammatory effects of surfactant and especially its lipid components on EC are unknown.

We recognize that the putative AEC-derived soluble factor(s) may only partly be lipid in nature, as lipids commonly exist in complex arrangements with other macromolecules (e.g. lipo-proteins, surfactant). Clearly, a complete evaluation of all potential AEC-derived lipid mediators is beyond the scope of this study, but should be systematically evaluated in future studies. Moreover, based on the normal close apposition of PMVEC and AEC in the normal alveolo-capillary barrier in vivo, AEC may also modulate PMVEC responses through direct AEC-PMVEC contact and other signalling mechanisms different from our putative AEC-derived soluble factor(s). For example, the presence of AEC in co-culture down-regulated endothelial cell E-selectin expression following LPS stimulation [36]. ± There are multiple pathways of PMVEC barrier dysfunction, although the exact mechanisms of septic PMVEC dysfunction and hyper-permeability have not been identified [37], [38], [39], [40]. Potential mechanisms include inter-cellular gap formation due to either RhoA-ROCK or myosin light chain kinase-dependent PMVEC retraction or disruption of PMVEC adherens and/or tight junctions. Alternatively, septic PMVEC apoptotic death and shedding could also affect the pulmonary microvascular barrier. In addition, the presence and interaction of activated leukocytes, specifically PMNs, enhances ALI and PMVEC dysfunction, which could be mediated through PMN-PMVEC adhesion-dependent signalling, serine protease and metalloproteinase-dependent disassembly of adherens/tight junctions, or induction of PMVEC death [41], [42]. We have previously reported that septic PMVEC barrier dysfunction was highly dependent on PMN-PMVEC adhesion, and PMN iNOS expression/activity in both animal models and isolated human cells [9], [43].

In the present study, A549 cell-dependent attenuation of both PMN-dependent PMVEC permeability and trans-PMVEC PMN migration, but a lack of effect on PMN-PMVEC adhesion, is consistent with a mechanistic change in intra-PMVEC signalling rather than a simple change in PMVEC surface adhesion molecule expression. However, since we did not assess PMVEC permeability mechanisms, the putative AEC-dependent protective effect against septic PMVEC hyper-permeability could be mediated through inhibition of any of the possible mechanisms of septic PMVEC hyper-permeability described above.

We recognize other limitations of our study. Our simple in vitro experimental model cannot be entirely representative of the normal human alveolo-capillary unit, where PMVEC and AEC interact with multiple other cell types, including fibroblasts, dendritic cells, and inflammatory cells [26]. Moreover, septic ALI/ARDS at the bedside reflects alveolar flooding following leak of vascular plasma/albumin across the PMVEC, basement membrane, interstitial space, and AEC; in this study, we have only addressed trans-PMVEC albumin leak. The use of A549 cells as a model of human AEC is well accepted, but we recognize the limitations of such a cell line. However, we are reassured that the identified AEC-dependent protective mechanism was confirmed in a limited number of key studies with primary human AEC. Unfortunately, given the difficulty in isolating these cells and the resulting limited number of primary human AEC, all studies could not be performed with these cells.

In conclusion, human AEC appear to be an important protective mechanism against septic PMVEC hyper-permeability, not just as a consequence of their physical barrier at the alveolar-capillary junction, but more importantly via production of unidentified soluble factor(s), which are likely at least partly lipid in nature. This mechanism suggests a scientific and potential clinical therapeutic importance of epithelial–endothelial cross talk in maintaining alveolar integrity in ALI/ARDS. Future studies will better define the soluble factor(s) responsible for PMVEC protection, as well as explore the therapeutic potential of this epithelial-endothelial interaction.

## References

[1] Tomashefski JF (2000) Pulmonary pathology of acute respiratory distress syndrome. Clin Chest Med 21: 435–466. 11019719 10.1016/s0272-5231(05)70158-1

[2] Matthay MA, Zemans RL (2011) The acute respiratory distress syndrome: pathogenesis and treatment. Annu Rev Pathol 6: 147–163. 20936936 10.1146/annurev-pathol-011110-130158 PMC3108259

[3] Ware LB, Matthay MA (2000) The acute respiratory distress syndrome. N Engl J Med 342: 1334–1349. 10793167 10.1056/NEJM200005043421806

[4] Rubenfeld GD, Herridge MS (2007) Epidemiology and outcomes of acute lung injury. Chest 131: 554–562. 17296661 10.1378/chest.06-1976

[5] Razavi HM, Wang L, Weicker S, Quinlan GJ, Mumby S, et al (2005) Pulmonary oxidant stress in murine sepsis is due to inflammatory cell nitric oxide. Crit Care Med 33: 1333–1339. 15942352 10.1097/01.ccm.0000165445.48350.4f

[6] Razavi HM, Wang LF, Weicker S, Rohan M, Law C, et al (2004) Pulmonary neutrophil infiltration in murine sepsis: role of inducible nitric oxide synthase. Am J Respir Crit Care Med 170: 227–233. 15059787 10.1164/rccm.200306-846OC

[7] Wang LF, Patel M, Razavi HM, Weicker S, Joseph MG, et al (2002) Role of inducible nitric oxide synthase in pulmonary microvascular protein leak in murine sepsis. Am J Respir Crit Care Med 165: 1634–1639. 12070065 10.1164/rccm.2110017

[8] Bhatia M, Moochhala S (2004) Role of inflammatory mediators in the pathophysiology of acute respiratory distress syndrome. J Pathol 202: 145–156. 14743496 10.1002/path.1491

[9] Shelton JL, Wang L, Cepinskas G, Sandig M, Scott JA, et al (2007) Inducible NO synthase (iNOS) in human neutrophils but not pulmonary microvascular endothelial cells (PMVEC) mediates septic protein leak in vitro. Microvasc Res 74: 23–31. 17451752 10.1016/j.mvr.2007.02.008

[10] Shelton JL, Wang L, Cepinskas G, Sandig M, Inculet R, et al (2006) Albumin leak across human pulmonary microvascular vs. umbilical vein endothelial cells under septic conditions. Microvasc Res 71: 40–47. 16376951 10.1016/j.mvr.2005.11.003

[11] Farley KS, Wang LF, Razavi HM, Law C, Rohan M, et al (2006) Effects of macrophage inducible nitric oxide synthase in murine septic lung injury. Am J Physiol Lung Cell Mol Physiol 290: L1164–L1172. 16414981 10.1152/ajplung.00248.2005

[12] Gorin AB, Stewart PA (1979) Differential permeability of endothelial and epithelial barriers to albumin flux. J Appl Physiol 47: 1315–1324. 536303 10.1152/jappl.1979.47.6.1315

[13] Berthiaume Y, Matthay MA (2007) Alveolar edema fluid clearance and acute lung injury. Respir Physiol Neurobiol 159: 350–359. 17604701 10.1016/j.resp.2007.05.010 PMC2682357

[14] Manicone AM (2009) Role of the pulmonary epithelium and inflammatory signals in acute lung injury. Expert Rev Clin Immunol 5: 63–75. 19885383 10.1586/177666X.5.1.63 PMC2745180

[15] Thorley AJ, Ford PA, Giembycz MA, Goldstraw P, Young A, et al (2007) Differential regulation of cytokine release and leukocyte migration by lipopolysaccharide-stimulated primary human lung alveolar type II epithelial cells and macrophages. J Immunol 178: 463–473. 17182585 10.4049/jimmunol.178.1.463

[16] Witherden IR, Vanden Bon EJ, Goldstraw P, Ratcliffe C, Pastorino U, et al (2004) Primary human alveolar type II epithelial cell chemokine release: effects of cigarette smoke and neutrophil elastase. Am J Respir Cell Mol Biol 30: 500–509. 15033639 10.1165/rcmb.4890

[17] Wang L, Bastarache JA, Wickersham N, Fang X, Matthay MA, et al (2007) Novel role of the human alveolar epithelium in regulating intra-alveolar coagulation. Am J Respir Cell Mol Biol 36: 497–503. 17099142 10.1165/rcmb.2005-0425OC PMC1899324

[18] Ehrhardt C, Kim KJ, Lehr CM (2005) Isolation and culture of human alveolar epithelial cells. Methods Mol Med 107: 207–216. 15492374 10.1385/1-59259-861-7:207

[19] Hermanns MI, Fuchs S, Bock M, Wenzel K, Mayer E, et al (2009) Primary human coculture model of alveolo-capillary unit to study mechanisms of injury to peripheral lung. Cell Tissue Res 336: 91–105. 19238447 10.1007/s00441-008-0750-1

[20] Bone RC, Sibbald WJ, Sprung CL (1992) The ACCP-SCCM consensus conference on sepsis and organ failure. Chest 101: 1481–1483. 1600757 10.1378/chest.101.6.1481

[21] Schnitzer SE, Weigert A, Zhou J, Brüne B (2009) Hypoxia enhances sphingosine kinase 2 activity and provokes sphingosine-1-phosphate-mediated chemoresistance in A549 lung cancer cells. Mol Cancer Res 7: 393–401. 19240180 10.1158/1541-7786.MCR-08-0156

[22] Singh LS, Berk M, Oates R, Zhao Z, Tan H, et al (2007) Ovarian cancer G protein-coupled receptor 1, a new metastasis suppressor gene in prostate cancer. J Natl Cancer Inst 99: 1313–1327. 17728215 10.1093/jnci/djm107

[23] Shelton JL, Wang L, Cepinskas G, Sandig M, Scott JA, et al (2007) Inducible NO synthase (iNOS) in human neutrophils but not pulmonary microvascular endothelial cells (PMVEC) mediates septic protein leak in vitro. Microvasc Res 74: 23–31. 17451752 10.1016/j.mvr.2007.02.008

[24] Moss M, Gillespie MK, Ackerson L, Moore FA, Moore EE, et al (1996) Endothelial cell activity varies in patients at risk for the adult respiratory distress syndrome. Crit Care Med 24: 1782–1786. 8917025 10.1097/00003246-199611000-00004

[25] Ware LB, Eisner MD, Thompson BT, Parsons PE, Matthay MA (2004) Significance of von Willebrand factor in septic and nonseptic patients with acute lung injury. Am J Respir Crit Care Med 170: 766–772. 15201135 10.1164/rccm.200310-1434OC

[26] Clerici C (2011) The challenge of modeling human acute respiratory distress syndrome: a new model of lung injury due to sepsis with impaired alveolar edema fluid removal. Am J Physiol Lung Cell Mol Physiol 301: L20–L22. 21551081 10.1152/ajplung.00126.2011

[27] Shelton JL, Wang L, Cepinskas G, Inculet R, Mehta S (2008) Human neutrophil-pulmonary microvascular endothelial cell interactions in vitro: differential effects of nitric oxide vs. peroxynitrite. Microvasc Res 76: 80–88. 18616952 10.1016/j.mvr.2008.06.001

[28] Burns AR, Smith CW, Walker DC (2003) Unique structural features that influence neutrophil emigration into the lung. Physiol Rev 83: 309–336. 12663861 10.1152/physrev.00023.2002

[29] Thorley AJ, Goldstraw P, Young A, Tetley TD (2005) Primary human alveolar type II epithelial cell CCL20 (macrophage inflammatory protein-3alpha)-induced dendritic cell migration. Am J Respir Cell Mol Biol 32: 262–267. 15618437 10.1165/rcmb.2004-0196OC

[30] Mason RJ (2006) Biology of alveolar type II cells. Respirology 11 Suppl: S12–S15 10.1111/j.1440-1843.2006.00800.x 16423262

[31] Singleton PA, Dudek SM, Chiang ET, Garcia JG (2005) Regulation of sphingosine 1-phosphate-induced endothelial cytoskeletal rearrangement and barrier enhancement by S1P1 receptor, PI3 kinase, Tiam1/Rac1, and alpha-actinin. FASEB J 19: 1646–1656. 16195373 10.1096/fj.05-3928com

[32] Jacobs ER, Zeldin DC (2001) The lung HETEs (and EETs) up. Am J Physiol Heart Circ Physiol 280: H1–H10. 11123211 10.1152/ajpheart.2001.280.1.H1

[33] Bastarache JA, Fremont RD, Kropski JA, Bossert FR, Ware LB (2009) Procoagulant alveolar microparticles in the lungs of patients with acute respiratory distress syndrome. Am J Physiol Lung Cell Mol Physiol 297: L1035–L1041. 19700643 10.1152/ajplung.00214.2009 PMC2793184

[34] Wright JR (2005) Immunoregulatory functions of surfactant proteins. Nat Rev Immunol 5: 58–68. 15630429 10.1038/nri1528

[35] Orgeig S, Hiemstra PS, Veldhuizen EJ, Casals C, Clark HW, et al (2010) Recent advances in alveolar biology: evolution and function of alveolar proteins. Respir Physiol Neurobiol 173 Suppl: S43–S54 10.1016/j.resp.2010.04.023 PMC4097100 20433956

[36] Weppler A, Rowter D, Hermanns I, Kirkpatrick CJ, Issekutz AC (2006) Modulation of endotoxin-induced neutrophil transendothelial migration by alveolar epithelium in a defined bilayer model. Exp Lung Res 32: 455–482. 17169854 10.1080/01902140601059463

[37] Breslin JW, Yuan SY (2004) Involvement of RhoA and Rho kinase in neutrophil-stimulated endothelial hyperpermeability. Am J Physiol Heart Circ Physiol 286: H1057–H1062. 14630629 10.1152/ajpheart.00841.2003

[38] Pober JS, Min W, Bradley JR (2009) Mechanisms of endothelial dysfunction, injury, and death. Annu Rev Pathol 4: 71–95. 18754744 10.1146/annurev.pathol.4.110807.092155

[39] Perl M, Lomas-Neira J, Venet F, Chung CS, Ayala A (2011) Pathogenesis of indirect (secondary) acute lung injury. Expert Rev Respir Med 5: 115–126. 21348592 10.1586/ers.10.92 PMC3108849

[40] Ochoa CD, Stevens T (2012) Studies on the cell biology of interendothelial cell gaps. Am J Physiol Lung Cell Mol Physiol 302: L275–L286. 21964402 10.1152/ajplung.00215.2011 PMC3289273

[41] DiStasi MR, Ley K (2009) Opening the flood-gates: how neutrophil-endothelial interactions regulate permeability. Trends Immunol 30: 547–556. 19783480 10.1016/j.it.2009.07.012 PMC2767453

[42] Grommes J, Soehnlein O (2011) Contribution of neutrophils to acute lung injury. Mol Med 17: 293–307. 21046059 10.2119/molmed.2010.00138 PMC3060975

[43] Wang L, Taneja R, Razavi HM, Law C, Gillis C, et al (2012) Specific Role of Neutrophil Inducible Nitric Oxide Synthase in Murine Sepsis-Induced LungInjury in vivo. Shock 37: 539–547. 22392143 10.1097/SHK.0b013e31824dcb5a

